# Ulcerating Surfaces and Abscesses

**Published:** 1881-01

**Authors:** 


Ulcerating Surfaces and Abscesses.—In ulcerating surfaces and abscesses which show no tendency to close, the treatment must be directed toward the production of healthy granulations. This is often difficult to accomplish, and various applications are employed to stimulate the surface. Balsam of Peru is used largely in the hospital for this purpose. Where this does not succeed, the following combination is usually successful:

R.—Hydrargyri nitratis............gr. xx

Iodoformi.................gr. xxx

Camphoræ......................ℨij

Balsam Peru......................℥ij

M.—Sig. Stimulate balsam.

Chloral hydrate may be substituted for the camphor in the mixture. This is applied to the surface or injected into the abscess after the latter has been thoroughly washed out with carbolized water once a day, and rarely fails to stimulate the growth of healthy granulation tissue. When œdema sets in around a wound, and there is danger of cellulitis, the part is kept moist by frequent application of lint wet with lead and opium wash, as this is more efficacious than any other lotion. The proportions of the lotion are one ounce of tincture of opium to half an ounce of subacetate of lead in half pint of water.—*Chicago Medical Review.*

